# What is the effectiveness of obesity related interventions at retail grocery stores and supermarkets? —a systematic review

**DOI:** 10.1186/s12889-016-3985-x

**Published:** 2016-12-28

**Authors:** Abdulfatah Adam, Jørgen D Jensen

**Affiliations:** Department of Food and Resource Economics, University of Copenhagen, Rolighedsvej 25, DK-1958 Frederiksberg, Denmark

**Keywords:** Obesity intervention, Healthy foods, Food store, Supermarket, Review

## Abstract

**Background:**

The Prevalence of obesity and overweight has been increasing in many countries. Many factors have been identified as contributing to obesity including the food environment, especially the access, availability and affordability of healthy foods in grocery stores and supermarkets. Several interventions have been carried out in retail grocery/supermarket settings as part of an effort to understand and influence consumption of healthful foods. The review’s key outcome variable is sale/purchase of healthy foods as a result of the interventions. This systematic review sheds light on the effectiveness of food store interventions intended to promote the consumption of healthy foods and the methodological quality of studies reporting them.

**Methods:**

Systematic literature search spanning from 2003 to 2015 (inclusive both years), and confined to papers in the English language was conducted. Studies fulfilling search criteria were identified and critically appraised. Studies included in this review report health interventions at physical food stores including supermarkets and corner stores, and with outcome variable of adopting healthier food purchasing/consumption behavior. The methodological quality of all included articles has been determined using a validated 16-item quality assessment tool (QATSDD).

**Results:**

The literature search identified 1580 publications, of which 42 met the inclusion criteria. Most interventions used a combination of information (e.g. awareness raising through food labeling, promotions, campaigns, etc.) and increasing availability of healthy foods such as fruits and vegetables. Few used price interventions. The average quality score for all papers is 65.0%, or an overall medium methodological quality. Apart from few studies, most studies reported that store interventions were effective in promoting purchase of healthy foods.

**Conclusion:**

Given the diverse study settings and despite the challenges of methodological quality for some papers, we find efficacy of in-store healthy food interventions in terms of increased purchase of healthy foods. Researchers need to take risk of bias and methodological quality into account when designing future studies that should guide policy makers. Interventions which combine price, information and easy access to and availability of healthy foods with interactive and engaging nutrition information, if carefully designed can help customers of food stores to buy and consume more healthy foods.

**Electronic supplementary material:**

The online version of this article (doi:10.1186/s12889-016-3985-x) contains supplementary material, which is available to authorized users.

## Background

Several studies have indicated one of the main causes of obesity to be an environment that promotes excessive food intake and discourages physical activity [1–10]. Retail food stores and supermarkets are important environmental settings in this respect. Households in developed countries buy most of their food from retail groceries/supermarkets, and make an average of two visits to a supermarket per week [11, 12]. Several studies have shown that food stores, and the availability of products that are good for healthy living in those stores, are important contributors to healthy eating patterns among customers who frequent these stores [6, 9], and that grocery stores and supermarkets can play a unique role in helping to reverse the obesity epidemic [13, 14]. As a result, several interventions at food store level have been conducted to investigate this potential. Therefore, it is imperative to undertake a systematic review of these interventions and summarize the existing evidence. An overview of the research conducted in this area so far will be useful not only for researchers interested in healthy food consumption interventions, but the conclusions are also expected to assist policy makers in this area.

In this paper, we systematically review the literature on store-setting interventions aimed at increasing the consumption of healthy food (defined as foods whose consumption is recommended by expert bodies and national dietary guidelines [15, 16]), including the characteristics and effectiveness of the studied interventions as well as a methodological quality assessment of the research articles which meet the inclusion criteria.

In the past, some reviews that summarize evidence of the effectiveness of food store interventions on healthy food purchases have been published [9, 17–20]. However, these reviews are either old [19], limited in scope [9, 18], use narrative rather than systematic approach or lack rigorous assessment of the methodological quality of the studies surveyed [18]. Whereas the paper by Seymour et al. [19] looked at studies on “nutrition environmental interventions” dating between 1970 and 2003, we focus on the last decade, i.e., papers published between 2003 and 2015. Furthermore, although an important contribution in the area, the paper by Glanz et al. [20] used a narrative review approach, while ours differs in that we strengthen this by using a systematic review approach. The reviews by Gittelsohn et al. [18] and Escaron et al. [17] both synthesized the literature to investigate effectiveness of health interventions in store settings. While the scope of the former is limited to small-store interventions, the latter focused on consumption effects and included a broad range of store-setting interventions. Both papers found an overall intervention effect for obesity-related store interventions. According to Escaron et al. [17], this effect was even higher for interventions using a combination of strategies. Further, the authors noted a need for more rigorous interventions. Finally, a review surveying in-store interventions [9] solely focused on fruits and vegetables (F&V). Our review is similar to that of Gittelsohn et al. [18] and Escaron et al. [17] both of which looked at food store interventions aimed at promoting healthful food consumption behavior with the conclusion that the interventions improved healthy food choices. However, a novel contribution of our study is that, in addition to updating existing literature with recently published papers, we put methodological quality of studies to the test. This is important because the focus on food environment, and particularly in-store interventions, has been gaining ground recently, and important studies have been published since the last reviews. Despite including pricing as one of the possible store intervention strategies, studies using store-setting price incentives included in the past reviews have either been old [21, 22] or few [9]. Since then, some important studies on the effect of price incentives on food purchase in store settings have been published. In addition to assessing the contribution of the newly published papers [23–27], our review also includes studies not considered by previous reviews [28, 29]. In contrast to the previous review studies, we exclude grey literature, because we aim to establish the methodological quality. Similar arguments hold for our review’s contribution in comparison to the review by Liberato et al. [30].

## Methods

### Search strategy

The systematic review was conducted in accordance with the Preferred Reporting Items for Systematic Reviews and Meta-Analyses (PRISMA) statement [31]. For the literature search, PubMed, Google Scholar, and EconLit databases were used. These three databases have each their strengths. PubMed is one of the most used databases for searching health interventions, EconLit has its strengths with regard to the economic literature, and Google Scholar has a relatively broad general coverage within the academic literature. Keywords used to search for potential studies are provided in the supplementary material (Additional file 1 appendix 1). Extracted studies’ titles and abstracts were later screened against the inclusion criteria. Additional studies were identified by analysis of literature cited by retrieved papers.

### Inclusion/exclusion criteria

Only studies written in the English language and only peer-reviewed papers published between the years 2003 and 2015 (both years included) were included. The time frame was chosen to select research that provides recent evidence and reflects up-to-date conditions of store structure and modes of communication between retailers and customers. Our outcome of interest in this review is the adoption of healthier food purchasing. The review focuses on studies of retail food store interventions that are related to obesity, and has purchase/consumption effects as an outcome measure.

The scope of this review is limited to interventions intended to increase consumption of healthy food in store settings. Therefore, research which is primarily focused on marketing, e.g. East et al. [32] or Sigurdsson et al. [33], have been excluded. There is no consensus on the definitions of the terms healthy foods and unhealthy foods [34]; however, similar to Glanz and Yaroch [9], in this review we consider foods whose consumption is recommended by national diet guidelines, such as the American diet guidelines [16] and Danish diet guidelines [35], as healthy. Unhealthy foods refer to high energy density products and processed foods with no or low nutritional value. Furthermore, only studies which feature interventions carried out in actual physical retail food stores are considered. Retail food stores are defined as stores whose primary merchandise is food, but with different sales volumes and range of foods provided. They include grocery stores, supermarkets, and convenience stores with supermarkets having full range of food products and high annual gross sales (≥2 mio US dollars) while convenience stores are the opposite with limited shelf space and product range [36]. In other words, studies based on online grocery stores [37–39], in controlled settings such as laboratories [40] or at schools [41] are not included in the review. Finally, interventions where the promoted food is delivered to the home have also been excluded [42, 43], as they take place outside store settings.

Previous reviews [9, 17] have grouped grocery interventions into one of four rubrics: point-of-purchase (POP) information, pricing (affordability), increased availability of healthy foods, and promotion and advertising. In this paper, POP information & promotion and advertising are organized under the single heading of information. Therefore, the considered papers have one or more of the following three main intervention components: affordability (price), information and access/availability.

### Screening

Studies that were identified by the search databases were further screened by the first reviewer (AA). The initial screening was based on relevance of the identified studies’ title and abstract. Full text of those studies deemed to be potentially relevant for our review were retrieved (with the exception of two cases where we had to request the full text from the first author because the full text was either not available on the net or we had not access to it). AA assessed the relevance of the retrieved papers and these were later checked by the second reviewer (JDJ). The two reviewers were in agreement of the final list of the papers included in the review.

### Data extraction

The following information was extracted for 42 full text articles meeting the inclusion criteria: primary outcome of the study, study design, key findings, target group, country, type of intervention, and description of the intervention. To facilitate structure and organization of the review, each study was grouped under the three main intervention headings of price/affordability, increased accessibility/availability and information. Further, articles were subdivided into single intervention or multiple interventions based on the number of intervention strategies they adopted.

### Assessing the studies’ methodological quality and risk of bias

Despite the similar overall goal of the studies meeting our inclusion criteria, they often are diverse in terms of their study designs, data collection methods, type of data and analytical methods used. This complicates comparison of methodological quality across the studies. Moreover, although many quality assessment tools have been proposed [44], some are specific to certain study designs such as randomized controlled trials [45]. In order to take the broad nature of the studies into account, and to avoid bias towards quantitative methods, we use a transparent and validated tool developed by Sirriyeh et al. [46] and used by Vyth et al. [47] and Haugum et al. [48] among others. The tool consists of 16 criteria each with a score ranging between 0 and 3, with 3 being the best.

The 16 criteria reflect aspects of clarity in description of aims and setting, data quality, method of analysis and self-evaluation. For description of the 16 criteria, see supplementary material (Additional file 2 appendix 2). Fulfillment of each of the 16 criteria was assessed independently by the two authors (and subsequently consolidated by consensus) for each publication, based on the information provided in the assessed paper, and a score corresponding to the level of satisfactory attainment of the criteria as outlined by Sirriyeh et al. [46] was assigned. For each paper, the scores were added and divided by the maximum possible score to report the paper’s overall quality score. It should be noted that if authors have not included the level of detail required to make a judgement for a quality criterion, then a score of 0 is awarded for that criterion. Attempts were made to contact authors of included studies some of which were not fruitful. Initial data extraction and screening was done by the first author and was later validated by the second author.

This is supplemented by assessment of studies’ risk of bias in line with Cochrane guidelines [49] and PRISMA [31]. Criteria for risk of bias assessment included random allocation (in relation to stores, shoppers or both), risk of selection bias, blinding (either analysts, store customers or both), control of possible confounders via statistical modelling and a priori power calculation. Each study received a summary of risk of bias score (high, medium, or low) based on Cochrane taxonomy (see table 8.7a in the Cochrane Handbook [49]). Table 1 describes the assignment of the risk of bias score.

**Table 1:** Scores used to assess risk of bias

Risk of bias	Interpretation	Relationship to individual bias criteria
Low	Possible bias, unlikely to seriously affect the study results	All criteria met; if criteria not reported, study does not drop to medium category unless random/concealed allocation criteria not reported
Medium	Possible bias that raises some doubt about the results	One or more criteria partially met
High		One or more criteria not met

Adapted from The Cochrane Handbook [49]

## Results

A formal meta-analysis was not possible due to the heterogeneous nature of the studies’ settings, designs and outcome measures. Hence, studies with similar intervention components were grouped together for narrative synthesis.

### Characteristics of the included studies

**Fig. 1:**
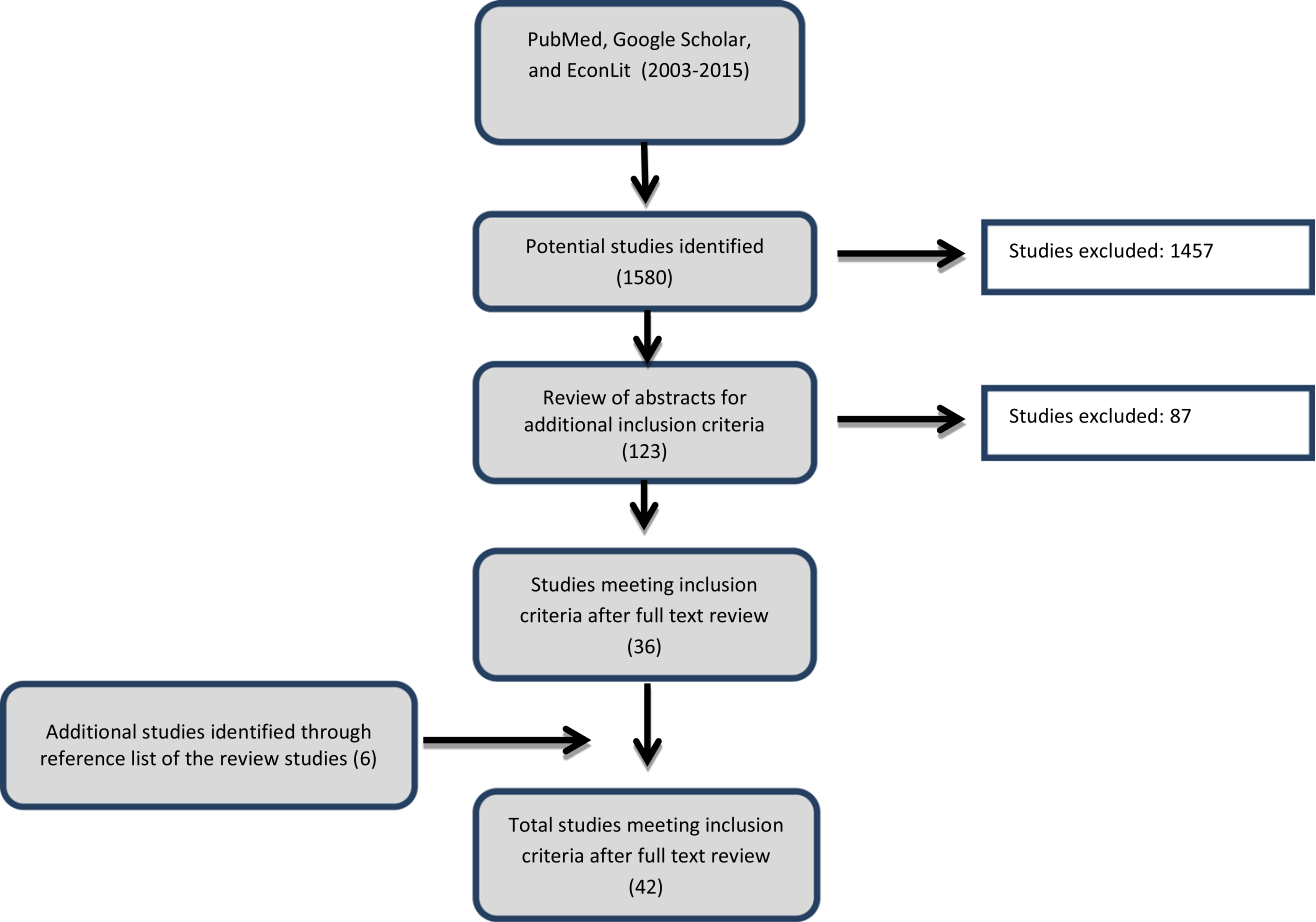
Flow chart of the Literature Review

During the search for relevant papers for inclusion in the review, a total of 1580 potential papers were identified. After going through the titles and abstracts, a total of 123 were selected for further screening. Of these, 36 articles met the inclusion criteria (Fig. 1). 6 additional studies were later identified via references and added to the analysis.

Table 2 presents a summary of the study characteristics. Data on study design, effectiveness, outcomes, etc., were summarized for studies that met the inclusion criteria. The last two columns of Table 2 summarize the result of the methodological quality (presented as a percentage of the maximum possible score) and risk of bias assessments.

**Table 2:** Summary of studies

Intervention Category	Country	Program/project name	Settings and Target group	Study Design	Outcome variable and targeted foods	Key Findings	QAT score (%)	Risk of bias
Information								
Milliron et al. (2012) [81]	U.S.A.	EatSmart	Urban supermarket; adult participants are targeted in a socioeconomically diverse region of Phoenix	Randomized controlled trial	Purchases of total, saturated, and trans fat (grams/1,000 kcal), and fruit, vegetables, and dark-green/yellow vegetables	The intervention positively affected purchase of fruit and dark-green/yellow vegetables. No other group differences were observed.	83.3%	medium
Sutherland et al. (2010) [77]	U.S.A.	Guiding Stars	168 supermarket stores in both rural and metropolitan areas	“Natural” experimental design	Sales of star-labelled foods before and after intervention	Sustained and significant changes in food purchasing after implementation and at follow-up reported	57.1%	high
Ogawa et al. (2011) [56]	Japan	-	Two urban supermarkets in two Japanese cities	pre-post study with control group	Sales of fruit and vegetables before and after intervention	Sales of fruit and vegetables of all types significantly increased during the intervention period at intervention store.	42.9%	high
Steenhuis et al. (2004) [63]	The Netherlands	-	Clients in 13 urban supermarkets were targeted	randomized, pre-post, experimental control group design	Fat intake	The education intervention, neither in stand-alone nor when coupled with the labeling had no significant effects	89.6%	low
Steenhuis et al. (2004) [62]	The Netherlands	-	Conducted at Supermarkets and worksite cafeterias and target was their clients	Description of program history and phases	Fat intake	The findings suggest that programmes should be promoted intensively. Furthermore, the relevant manufacturers and Wholesalers supplying worksite cafeterias should be encouraged to increase their range of suitable low-fat products	57.1%	high
Colapinto and Malaviarachchi (2009) [52]	Canada	Paint Your Plate	17 grocery stores in the City of Greater Sudbury; adults with diverse socioeconomic status were targeted	Pre-post with a comparison group	Knowledge of fruit serving size	Intervention participants were six times more likely than participants receiving brochures to identify a serving size of fruit and vegetables; however, this difference vanished at follow-up	54.8%	high
Freedman and Connors (2010) [69]	U.S.A.	Eat Smart	Multi-ethnic college students shopping at on-campus convenience store	Quasi-experimental study	Sale specific promoted foods	Purchase of tagged food items increased	42.9%	high
Salmon et al. (2015) [64]	The Netherlands	Health on Impulse	127 customers of a Dutch supermarket	Cluster Randomized Controlled Trial	Sale of low calorie cheese	Nudging ego-depleted consumers to purchase low fat cheese with the help of social proof is effective.	69.0%	medium
Prices								
Phipps et al. (2014) [25]	U.S.A.	-	Urban low-income supermarkets	Mixed-methods (longitudinal quantitative design supplemented with qualitative data)	Weekly purchases of targeted foods	Households sought out products with price discounts.	56.3%	medium
Geliebter et al. (2013) [24]	U.S.A.	Supermarket Discounts on Low-Energy Density Foods	Two urban supermarkets; overweight and obese adults with various demographic backgrounds were involved	Randomized controlled trial	Intake of fruit and vegetables (and BMI)	Discounts of low-energy density fruit and vegetables led to increased purchasing and intake of those foods	83.3%	medium
Herman et al. (2008) [28]	U.S.A.	Special Supplemental Nutrition Program for Women, Infants, and Children (WIC)	English or Spanish speaking WIC-recipient Women at 3 WIC sites	pre-post study with non-equivalent control-group design	Purchase of fruit and vegetables	Increase of consumption of fruit and vegetables by intervention participants; this increase was sustained at 6 months follow-up.	70.8%	medium
Herman et al. (2006) [29]	U.S.A.	Special Supplemental Nutrition Program for Women, Infants, and Children (WIC)	Low-income women, infants and children participating WIC program in suburban Los Angeles	pre-post study with non-equivalent control-group design	Fruit and vegetable purchases	Monetary incentives as a supplement to WIC had positive effect on fruit and vegetable purchase by low-income women participating the intervention.	50.0%	high
An et al. (2013) [23]	South Africa	Healthy Foods Benefit	Households that are members of South Africa’s largest private insurance company receive discounts on healthy foods at 800 participating supermarkets	Cohort study	Sale of healthy foods identified by Discovery Insurance Panel	Discounts for program participants increased consumption of healthy foods	78.6%	medium
Access and availability								
Cummins et al. (2005) [55]	U.K.	-	New Urban superstore in socially underserved area; study participants were men and women aged 16 and above	Quasi-experimental design	Fruit and vegetable consumption in portions per day, psychological health	Positive effect on psychological health for intervention participants. No intervention effect on fruit and vegetable consumption.	73.8%	medium
Information and Price								
Mhurchu et al. (2007) [61]	New Zealand	The Supermarket Healthy Options Project (SHOP)	Target was main household shoppers at an urban supermarket in New Zealand	Randomized controlled trial	Purchase of fruit and vegetables.	Collection of electronic purchase data is a feasible way to assess effect of nutrition intervention on purchase behavior.	66.7%	medium
Mhurchu et al. (2010) [60]	New Zealand	The Supermarket Healthy Options Project (SHOP)	Supermarkets in urban Wellington; target group were Maori, Pacific, and non-Maori/ Non-Pacific ethnic groups	Randomized controlled trial	Change from baseline in percentage energy from saturated fat contained in supermarket food purchases at the completion of the 6-month trial intervention phase	The intervention reported no significant discounts nor tailored nutrition education on nutrients purchased.	92.9%	low
Blakely et al. (2011) [59]	New Zealand	The Supermarket Healthy Options Project (SHOP)	Maori, Pacific, and European customers of a Supermarket in New Zealand who had handheld scanner system were targeted	Randomized controlled trial	Purchase of fruit and vegetables.	Price discounts were associated with healthy food purchasing.	81.0%	low
Bihan et al. (2012) [57]	France	-	Low-income adults undergoing health examinations at centers affiliated with French Social Security, and 22 compliant supermarkets	Randomized controlled trial	Fruit and vegetable intake	Both stand-alone advice and advice combined with fruit and vegetable (FV) vouchers increased FV servings/day, with the latter leading to slightly higher FV servings/day	81.0%	low
Waterlander et al. (2013) [26]	The Netherlands	-	4 Dutch supermarkets in rural areas and their adult customers with low socioeconomic status are targeted	Randomized controlled trial	Purchase of fruit and vegetables (in grams) by households	Price discounts combined with education significantly increases purchase of fruit and vegetable	83.3%	low
Ball et al. (2015) [27]	Australia	Supermarket Healthy Eating for Life (SHELf)	574 women customers of an Australian supermarket	Randomized Controlled Trial	Sale of F&V and beverages	Price reductions had a partial effect (i.e., on some of the targeted foods)	90.5%	low
Information AND Access/ availability								
Foster et al. (2014) [73]	U.S.A.	-	Urban low-income supermarkets	Cluster-randomised controlled trial	Weekly sales of targeted products	Placement strategies can significantly enhance the sales of healthier items in several food and beverage categories	76.2%	medium
Sigurdsson et al. (2014) [58]	Norway	-	A convenience store and a discount store; and healthy foods	Alternating treatment design	Sale of targeted healthy foods	Placing healthy food items at the store checkout can lead to a substantial impact on sales of these products.	47.6%	high
Kennedy et al. 2009 [50]	U.S.A.	Rolling Store	A flexible store in Louisiana targeting African American Women	Randomized controlled trial	Increase consumption of fruit and vegetables, and to prevent weight gain	Intervention participants showed a weight loss of 2.0 kg, whereas the control group gained 1.1 kg. But change in energy intake was not significant.	56.3%	high
Gittelsohn et al. (2006) [65]	The Republic of Marshall Islands	The Republic of Marshall Islands (RMI) Healthy Stores project	Stores in a developing country (RMI); target were nutritionally deprived communities in RMI	Pre-post pilot study	Fruit and vegetables, and other healthy foods such as foods with lower fat alternatives.	High levels of exposure to the intervention were achieved during the 10-week period of implementation.	56.3%	high
Curran et al. (2005) [71]	U.S.A.	Apache Health Stores	Ethnic minority (American Indians) facing healthy food access problems	Process evaluation: assess fidelity, dose, reach and context	Number of healthy foods stocked; and number in-store promotion activities	Intervention was implemented with a high level of dose and reach, and a moderate to high level of fidelity	52.4%	high
Gittelsohn et al. (2010) [75]	U.S.A.	Health Foods Hawaii	Five stores in two Low-income ethnic minority communities; children and mothers were particularly targeted	Pre-post randomized trial	HEI score, HEI grain score, and water consumption	Intervention increased consumption of targeted healthy foods by children; also improved healthy food knowledge among caregivers.	66.7%	medium
Novotny et al. (2011) [82]	U.S.A.	Health Foods Hawaii	Low-income ethnic minority in rural Hawai’i; children and mothers were particularly targeted	Randomized Controlled Trial	Exposure (Dose, reach, fidelity)	Relatively high fidelity, dose and reach of store intervention was achieved. Availability was a challenge. Stocking decisions are not always controlled by storeowners/managers.	66.7%	low
Gittelsohn et al. (2013) [78]	U.S.A.	Navajo Healthy Stores	Stores in Low-income ethnic minority with poor food environment	Custer randomized controlled trial	Consumption intention and purchase of targeted healthy foods, BMI	Intervention was associated with reduced overweight/obesity and improved obesity-related psychosocial and behavioral factors among those persons most exposed to the intervention	58.3%	medium
Bains et al. (2013) [51]	Canada	Healthy Foods North	Low-income ethnic minority in Arctic Canada; focus was on women of childbearing age	Cluster randomized controlled trial	Energy and selected nutrient intakes, nutrient density and dietary adequacy	The intervention had a positive effect on vitamin A and D intake by intervention participants. No significant impact on calorie, sugar, or fat consumption	64.3%	medium
Ho et al. (2008) [53]	Canada	The Zhiiwapenewin Akino’maagewin: Teaching to Prevent Diabetes (ZATPD)	Grocery stores in Remote communities in Canada and their low-income ethnic minority customers	Quasi-experimental pretest/posttest evaluation	Food-related behavioral and psychosocial outcomes	Reported significant change in knowledge among intervention participants. There was also a significant increase in frequency of healthy food acquisition among respondents in the intervention communities.	50.0%	high
Rosecrans et al.(2008) [54]	Canada	The Zhiiwapenewin Akino’maagewin: Teaching to Prevent Diabetes (ZATPD)	Grocery stores in Remote communities in Canada and their low-income ethnic minority customers	Assess fidelity, dose, reach and context	Number of foods promoted, number and content of promotion materials, etc.	Program implemented in- and out-of-store activities with moderate fidelity.	60.4%	high
Dannefer et al. (2012) [72]	U.S.A.	Healthy Bodegas	55 corner stores in underserved urban neighborhoods	Pre-post design	Number and type of foods stocked, etc.	Participating stores significantly improved healthy food inventory; also moderate increase customer purchase of healthy foods.	52.4%	high
Holmes et al. (2012) [80]	U.S.A.	Healthy Kids Campaign	Urban grocery store intervention targeting children and their parents	Observational time-series without comparison	Sale of fruit and vegetables	Sale of targeted foods including fruits and vegetables increased.	52.1%	high
Ayala et al. (2013) [66]	U.S.A.	*Vida Sana Hoy y Mañana* (Healthy Life Today and Tomorrow)	*Tiendas* in central North Carolina and targeted mainly Hispanic customers of the tiendas.	Cluster Randomized controlled trial	sale of fruit and vegetables	Moderate intervention effect in reported fruit and vegetable intake	70.8%	medium
Caldwell et al. (2008) [67]	U.S.A.	Colorado Healthy People 2010 Obesity Prevention Initiative.	Stores in Colorado and various target groups including, older adults, high-risk individuals, and general community members	Pre-post study design	Fruit and vegetable intake	Significant increase in consumption of fruit and vegetables by intervention participants	61.9%	high
Martínez-Donate et al. (2015) [79]	U.S.A.	Waupaca Eat Smart (WES)	601 customers at intervention & control supermarkets	Randomized Community trial	Reach, fidelity; availability and sale of healthy foods such as F&V	significant, but small improvements in the reported healthiness of target group purchases	60.4%	high
Price AND Access/ availability								
Andreyeva et al. (2012) [70]	U.S.A.	Special Supplemental Nutrition Program for Women, Infants, and Children (WIC)	Urban grocery store and supermarket intervention targeting women and infants	Pre-post study	Fruit and vegetables and variety of healthy foods in WIC-authorized convenience and grocery stores	Revised WIC food packages had a significant positive effect on availability and variety of healthy foods in WIC-authorized and (to a smaller degree) non-WIC convenience and grocery stores.	71.4%	medium
Freedman et al. (2011) [68]	U.S.A.	The Veggie Project	Farmers’ markets intervention targeting Boys and Girls Clubs in ethnically minority low-income areas in Nashville with limited healthy food retail outlet	Pre-post Study	Sales of targeted healthy foods	Intervention led to purchase of fresh fruit and vegetables by participants	64.6%	high
Access/availability AND Information AND Price								
Gittelsohn et al. (2010) [74]	U.S.A.	Baltimore Healthy Stores; BHS	Urban corner stores in low-income area in Baltimore City	Quasi- experimental design	Food-related behavioral and psychosocial outcomes	Overall healthy food purchasing scores, food knowledge, and self-efficacy did not show significant improvements associated with intervention status. But, intervention had a positive effect on healthiness of food preparation methods and showed a trend toward improved intentions to make healthy food choices	66.7%	high
Gittelsohn et al. (2010) [97]	U.S.A.	Baltimore Healthy Stores; BHS	Urban corner stores in low-income area in Baltimore City	Assess Reach, dose and fidelity	Number of foods promoted, number and content of promotion materials, number of discount coupons handed, etc.	Program implemented successfully in small and large stores in a low-income area of Baltimore City. Many lessons learned. The most important being that successful implementation of such a store-based program is feasible	61.9%	high
Song et al. (2011) [83]	U.S.A.	Baltimore Healthy Stores; BHS	Urban corner stores in low-income area in Baltimore City	Process evaluation (focus of storeowners perception)	storeowners’ perception of Baltimore Healthy Stores Intervention	The storeowners varied significantly in their level of acceptance and participation in the program. Strong and moderate support storeowners had a more positive attitude toward the community and the program.	54.8%	high
Song et al. (2009) [76]	U.S.A.	Baltimore Healthy Stores; BHS	Urban corner stores in low-income area in Baltimore City	Quasi- experimental design	Sale of targeted healthy foods	Significant intervention increase in sales of some promoted healthy foods, compared to comparison group.	60.4%	high

All of the included papers were on grocery interventions that aimed at increasing the consumption of healthy foods. Most papers were research reports of larger programs/projects. The table summarizes the programs reported and the connected articles

The studies are very diverse in terms of study design, method of data collection, sample size, and target population. The study sample sizes range from 37 supermarket customers [50] to more than 200,000 beneficiaries of a large intervention [23]. Most studies were conducted in the U.S.A. Four were conducted in Canada [51–54], one in the UK [55], one in Japan [56], one in France [57], one in South Africa [23], one in Norway [58], one in Australia [27] three in New Zealand [59–61], four in the Netherlands [26, 62–64] and one in the Republic of Marshal Islands [65].

Fruits and vegetables (F&V) were targeted by the majority of the interventions as healthy foods [24, 26–29, 50, 52, 55, 57, 66–69]. In addition to F&V, several studies looked into other healthy foods [51, 65, 70–76]. Four studies considered effects of interventions on both healthy and unhealthy foods [25, 59, 60, 77]. Finally, one study [58] focused on dried fish and fruit mix, while others [27, 63, 64] targeted low-calorie products.

Fifteen studies use quasi-experimental designs [28, 29, 52, 53, 55, 56, 65, 67–70, 72, 74–76], while twelve utilize randomized/cluster-randomized study designs [24, 26, 27, 57, 59, 60, 63, 64, 66, 73, 78, 79]. Moreover, most use self-reported data or dietary recalls, but some of the studies used electronic sales data [26–28, 56, 58–60, 68, 69, 73, 77, 80].

### Methodological quality and risk of bias of included studies

According to the chosen assessment tool, the methodological quality scores of the papers range from lowest score of 42.9% to highest score of 92.9%, yielding an average quality score for all papers of 65.0%. Most of the studies with scores higher than 80% are studies with randomized controlled trials [26, 27, 57, 59, 60, 63, 78, 81]. Criteria for which most studies scored low, as a percentage of the total possible score (100%), included assessment of reliability of analytic process (33.3%), evidence of sample size considered in terms of analysis (29.4%), statistical assessment of reliability and validity of measurement tool(s) (34.2%), evidence of user involvement in design (42.1%), and good justification for analytic method selected (42.1%). Fit between stated research question and method of data collection was 68.3% and 75.4% for quantitative and qualitative studies, respectively. Since most studies were concerned about testing effect of intervention, studies with randomized controlled trials generally scored high on this criterion.

The result contained in Table 3 shows mean and standard deviation of the 16 methodological assessment criteria. According to this table, almost all evaluated studies get the maximum possible score of 3 for “clear description of research setting”, “statement of aims/objectives in main body of report” and “description of procedure for data collection”. The lowest average scores are found for criteria “evidence of sample size considered in terms of analysis”, “Statistical assessment of reliability and validity of measurement tool(s) (quantitative only)” and “assessment of reliability of analytic process (qualitative only)” for which the studies received an average score of 0.88, 1.03 and 1.00, respectively.

**Table 3:** List of the 16 criteria used to assess the methodological quality of the studies included in the review

#	Criteria	Mean	S.D.^*^	#	Criteria	Mean	S.D.^*^
1	Explicit theoretical framework	1.88	0.77	9	Statistical assessment of reliability and validity of measurement tool(s) (Quantitative only)	1.03	1.12
2	Statement of aims/objectives in main body of report	2.93	0.26	10	Fit between stated research question and method of data collection (Quantitative only)	2.05	0.75
3	Clear description of research setting	2.98	0.15	11	Fit between stated research question and format and content of data collection tool e.g. interview schedule (Qualitative only)	2.26	0.65
4	Evidence of sample size considered in terms of analysis	0.88	1.23	12	Fit between research question and method of analysis (Quantitative only)	2.28	0.64
5	Representative sample of target group of a reasonable size	1.69	0.72	13	Good justification for analytic method selected	1.26	1.01
6	Description of procedure for data collection	2.93	0.26	14	Assessment of reliability of analytic process (Qualitative only)	1.00	0.88
7	Rationale for choice of data collection tool(s)	2.17	0.79	15	Evidence of user involvement in design	1.29	1.17
8	Detailed recruitment data	1.95	1.06	16	Strengths and limitations critically discussed	2.29	0.97

# stands for criteria number; ^*^S.D. is short for standard deviation

Most studies scored high to medium risk of bias (see last column of Table 2). Only seven studies have low risk of bias [26, 27, 57, 59, 60, 63, 82]. It is noteworthy that all of the latter studies are randomized controlled studies.

### Intervention types and key findings

Most of the interventions focused on increase in sales of healthy foods. Examples of healthy foods targeted include whole grains, F&V, lower-fat milk [23, 27, 70], healthier beverages, lower sugar cereals [73, 78], low-calorie beverages [27], vitamins A & D, calcium [51] and fish [58].

Apart from few studies [55, 59, 60, 62, 63, 74], store interventions have been found to be effective in one or more of their main outcomes. In some studies, overall energy intake did not significantly change [50], although positive and significant change in targeted food was achieved. Next we categorize articles into two, based on the number of intervention strategies they adopted.

#### Single strategy interventions

We examined the studies based on whether they employed single intervention strategy or a combination of two or more. We first consider studies with single intervention strategies. Only one paper falls under the single-component intervention strategy of ***increased accessibility/availability*** [55]. The authors report intervention results based on a quasi-experimental controlled before-and-after study in the UK based on the opening of a supermarket in an area previously lacking a retail infrastructure. They found that this intervention had no significant effect on customers regarding the consumption of fruit and vegetables compared to a control group.

Five studies [23–25, 28, 29] that met the inclusion criteria used a stand-alone ***price/affordability intervention*** strategy. All of these interventions targeted foods that are related to health outcomes, mainly F&V [23–26, 28, 29], but also whole grains [23], bottled water, and diet sodas [24] and low calorie foods [25]. The used price interventions were in the form of vouchers worth US $10/week for F&V [28, 29], 50% discount on F&V and other healthy foods [24], 25% discount on selected healthy food items [23], and varied price reductions on low-calorie foods [25]. All five concluded that price reductions had a positive effect on the purchase and consumption of healthy food. The results indicate that the higher the discount the higher and more significant the intervention effect pointing to positive dose-response effect of price interventions.

Studies with an ***information*** intervention alone had the information displayed in the form of shelf and product labels, posters, flyers, and the distribution of educational brochures [52, 56, 63, 64, 69, 77, 81]. Three of these [56, 69, 77] found an increase in sales for the promoted food items. While the study by Milliron et al. [81] found an effect for the outcome of F&V purchase, Steenhuis et al. [63] found no intervention effect, and Colapinto and Malaviarachchi [52] could not see a sustained effect at follow-up.

#### Multi-component interventions

Among the studies that met the inclusion criteria, 14 have ***combined information and access/availability*** elements [50, 51, 53, 58, 65–67, 72, 73, 75, 78–80, 82]. All of these studies reported positive effect in one or more of their outcome measures, particularly, increase in purchase of healthier foods. One project not only increased healthy food purchase, but also reported weight loss for participating individuals [50]. Some of the studies which combined components of interventions on information and availability also included aspects other than nutrition/food. For instance, Bains et al. [51] incorporated physical activity alongside the component targeting retail grocery shops. Other prevention programs used multiple settings and not just grocery stores [53, 79].

Five papers reported interventions based on a ***combined monetary incentives and information*** [26, 27, 57, 59–61]. A French randomized controlled trial (RCT) found that face-to-face group dietary advice from a trained dietician combined with discounts had a stimulating effect on the consumption of fruit and vegetables amongst intervention participants [57]. A similar randomized controlled trial in Dutch supermarkets in rural areas showed that nutrition education in the form of telephone counseling and provision of recipe books combined with price discounts had a significantly positive effect on the consumption of fruit and vegetables [26]. On the other hand, two New Zealand studies found no evidence for price discounts and healthy food purchasing [60], even when ethnic differences are accounted for [59]. An Australian RCT combining skills based training with price incentives found partial effect for prices in that price reductions led to increase in purchase of some of the targeted healthy foods such as fruits [27].

Two studies refer to programs that address a mix of ***affordability and availability*** of healthy foods at store settings [68, 70]. The former documents the effect of a revised Women, Infants and Children (WIC) program in the U.S.A., which is a subsidy program for low-income mothers and children. The revision meant improved availability and variety of healthy foods in WIC-authorized stores which the authors assume translates to increased consumption of subsidized healthy foods for WIC participants [70]. The latter study concluded that the Farmers’ Market intervention led to increased access and purchase of fruit and vegetables by project participants [68].

Finally, three papers reported results from the same program: the Baltimore Healthy Stores (BHS), which used a combination of ***all the three intervention types*** [74, 76, 83]. This intervention is associated with higher sales and the increased availability of some promoted foods (low-sugar cereals, low-salt crackers & cooking spray) [76]. Gittelsohn et al. [74] reported an increase in purchase of promoted foods at intervention stores. Song et al. [83] reported similar results, but also described some of the challenges faced during project implementation including unforeseen conflicts among intervention partners and lack of sustained support from store owners.

#### Characteristics of effective interventions

Effectiveness of health interventions in increasing sales of healthful foods at food stores depends on several factors: type and number of intervention components employed, incentive structure (e.g. WIC [70] or Vitality HealthyFood program [23]), stakeholder involvement and approval, community/consumer engagement, and depth of intervention implementation [26, 63].

The one component that people respond most strongly to seems to be the economic incentive (an exception being the study by Mhurchu et al. [60]), with its many forms: direct price discounts [26], vouchers for healthy foods [57], or subsidies of certain nutritious foods [23, 70]. Especially vouchers are worthy of further investigation [57], as vouchers have the advantage of forcing the consumer to buy only the food tied to the voucher (e.g., F&V) [28, 57]. Most pricing studies in this review have a subsidy nature, because they either offer vouchers or price discounts on healthy foods. The law of demand predicts consumers’ anticipated responses to price reductions, but this response is further accelerated by observed higher price of healthy foods [84]. Moreover, marketing studies reported that not only price decrease, but also the depth of price reduction matters [85]. This also seems to be the case in the studies included in our review. Nevertheless, price changes may be difficult to implement, especially if their implementation is not cost-neutral, as someone has to finance the price cuts. In certain cases storeowners may be convinced that due to economies of scale they will not incur losses despite price reductions of healthier foods [66].

Interventions could be divided into large-scale and small-scale interventions based on affected population size. Effective large size interventions include WIC targeting low-income women and children in the United States [70] and the National discount program in South Africa [23], which targets households that are member of an insurance company and offers them a discount of up to 25% on healthier foods at more than 800 supermarkets throughout South Africa [23]. Due to their large scale, both these programs create incentives for supermarkets as well as targeted consumer groups to show pro-health behavior. Interestingly, in the case of the revised WIC intervention, not only did WIC-approved stores increase availability of healthy foods but also non-WIC food stores increased their stocking of certain healthy foods [70], although it may be debated whether this parallel increase in non-WIC stores is a spill-over of WIC intervention effect or a common trend. Small scale interventions were typically a pilot [50, 61] or have been based on single or few supermarkets [52, 55, 56, 69, 74, 75, 78, 80–82], and their effects tend to be mixed due to the variations in both settings and strategies implemented.

Apart from the size of population and intervention components employed, interventions that increased sale and consumption of healthful foods can also be categorized according to targeted population, e.g. ethnic, minority or rural populations, which tend to pursue relatively unhealthy food consumption patterns [3, 86–88]. Several of the studies have focused on access and availability of healthy foods target ethnic and minority groups [65, 75, 78, 82], finding that interventions targeted at minority groups have increased access to and availability of healthier foods as well as purchase of these foods by target groups, whereas effects of interventions in urban and mixed ethnicity settings were small or negligible. Common to these interventions was the use of diverse yet culturally tailored media campaigns and how they engaged the target groups with activities such as taste tests. Certain target groups (e.g. women and children) seem to respond more positively to food store based health interventions regardless of ethnicity and geographical location [51, 53, 65, 67, 68, 75, 80]. Changing behavior of women and children is of paramount importance since most food-at-home is cooked by women in many societies [28, 50, 53, 78], and because childhood habits (including eating lifestyle) play an important role on later life habits.

In contexts where availability of healthy food is an issue, such as remote areas inhabited by ethnic minorities, involvement of local producers and distributors in interventions has been found to be important for long term sustained intervention effect [82]. Using trained community members is helpful in intervention implementation and for the likelihood of project success [50, 51].

Studies identified storeowners’ attitude and level of cooperation as a critical factor for intervention success [72, 73]. Many storeowners have concerns over possible loss in profits due to health interventions [76, 89]. Storeowners’ concerns are, however, not always based on correct predictions, as shown by one study, where research staff was able to convince local storeowners that the store would be able to sell ready-to-eat F&V at a profit [66]. Storeowners could also be made aware of healthy alternatives to the unhealthy foods usually stocked near checkout area [58]. Incentives, both monetary [83] and material support [66, 76], and cultural and ethnic considerations may help motivate storeowners to implement health interventions, for example by employing research staff with similar cultural and language background as the storeowners [66, 72, 76, 78, 83].

In addition to storeowners, consumers are very important stakeholders for long-term success of interventions. In principle, consumers have the power to influence what is being sold in food stores through their demand, and if interventions can convince ordinary consumers to choose healthy foods, it is possible to ensure sustainability of the interventions. It seems that engaging consumers, in addition to the posters and shelf labels, is more helpful than mere labels or nutrition information [63]. Examples of successful consumer engagement include cooking demonstrations/taste tests, and interactive education [50, 52, 65, 72, 75].

## Discussion

Our findings draw attention to the methodological quality of studies reporting in-store healthy food interventions. Strength of the used methodological assessment tool is that it enabled us to assess both quantitative and qualitative studies fairly. This is important because there has been a growing recognition of the benefits of including diverse types of evidence within systematic reviews [90]. Furthermore, it adopts a realist, pragmatic approach that is supported by Seale [91], and that is best suited to circumstances in which our review is being conducted [46]. From assessment of the methodological quality we found that only few of the included studies can be categorized as high quality studies from a methodological point of view (particularly those using randomized controlled trials [26, 57, 73]), as most of the studies are observational in nature, lack control groups, employ small sample size, or report conclusions based on short term intervention [52, 55, 56, 69, 80, 81]. All these suggest that there is room for improvement in future studies. This quality assessment may represent a lower-end estimate, if studies actually fulfilling some of the criteria listed in the assessment tool without explicitly reporting them in the publication due to, for example, journal space limitations, in which case a zero score has been assigned.

We have also attempted to identify some important characteristics for effective interventions. Our results on intervention effectiveness compares to a number of alternative reviews [17–19]. However, our review adds more recent papers and distinguishes itself in the methodological assessment of the studies reporting the interventions are included. Findings from the review suggest that in-store health interventions are generally effective in stimulating purchase and consumption of healthy foods, in that all but six studies [55, 59, 60, 62, 63, 74] showed increase in purchase of targeted healthy foods. It should however be noted that three of the studies reporting no intervention effects were of relatively good quality and low risk of bias. But as several other high-quality studies found an increase in sales of healthy foods as a result of the food store interventions, we still tend to conclude that health interventions at food stores work.

Looking at which components to target, we can conclude that promotion campaigns alone might not deliver the desired results [26, 52, 63]. Effectiveness could, however, be increased by combining it with other components [26, 50, 80], because different components can reinforce each other. For instance, nutrition knowledge (possibly with the help of the concept of nudging [64], nutrition programs that target low-income and minority groups or consumer engagement activities [50, 52, 72]) combined with affordability is more likely to induce people to buy a healthy food than nutrition knowledge alone [26].

Translating the results into obesity rate is challenging. Firstly, it should be noted that increased consumption of certain desirable foods would not necessarily lead to decline in obesity rate [92], (although they could have other health benefits, such as increased intake of certain vitamins in F&V). Secondly, although our primary outcome of interest is purchase (and consumption as secondary outcome) of healthy foods, we checked to see if studies also looked at changes in subjects’ body mass index (BMI). Only few studies explicitly attempted to link consumption with changes in BMI [23, 26, 28, 50, 53, 78], which makes direct comparison of health effects in the studies a challenge, and it is not generally clear whether increase in the purchase of healthy foods is followed by decline in the sale of unhealthy foods, as most studies do not use data that can show changes in total sales [52, 69, 72]. On the other hand, changes in BMI may not be immediate, hence, could not be captured by short term studies. As addressed in recent work by Glanz et al. [93], considerable work needs to be done on developing measures that are flexible and comprehensive enough to be applied across a variety of studies, yet act as a common measurement tool.

Our review has several significant policy implications, the most important of which is perhaps that food store health interventions generally work, especially if they combine multiple components. Price incentives appear to be a powerful supporting mechanism in such combinations. We believe our systematic review gives a much broader picture of both methodological qualities of studies and effective interventions than single studies. Furthermore, as shown in this review, more needs to be done to plan and execute successful health interventions at food store settings. Particularly policy makers should invest more in high-quality studies to establish clearly what, when and how effective interventions work. Even though high-quality studies are costly to conduct they are necessary for sound policy recommendations.

Although context-specific, some interventions may be more likely to have an effect on purchase and consumption of healthy foods at supermarkets. One challenge in in-store interventions is dissipation of effect after the intervention period has ended (Colapinto and Malaviarachchi [52]). For maximum and sustained effect, policy makers may pursue large-scale and long-term health intervention strategies with effective combinations of intervention components and with right incentives for both food suppliers and consumers, probably involving public-private partnership or private-private partnerships [23] .

Our review suggests that probability of success is correlated with the targeted group as greater effect is found for studies focusing on women and children; and this may also have a greater long term effect and other positive spillovers on society. We cannot, however, rule out the incentive structure used by the interventions targeting women and children may be a confounding factor for the observed effect. In fact, most interventions fail because one or more critical agents lack necessary incentives to participate. Our review shows price interventions with enough discount depth are promising, especially when combined with other strategies. But they are not without challenges. The biggest challenge is who finances the price gap? Storeowners may be reluctant to forego their profits for increased sales of healthy foods. As increasing consumption of healthy foods is in the interest of society, policy makes should consider ways to make business of healthy foods attractive to both consumers and retailers in order to maximize social welfare. Although a subsidy for healthy foods is an attractive policy option, the cost-effectiveness of such policies needs to be investigated. They should also be designed in ways that ensure compliance, for example, by tying the subsidy to the targeted foods.

Our review should be seen in light of several limitations. Firstly, only studies whose settings include brick and mortar food stores are considered. Although physical food stores account for large part of food sold to households, other points-of-sales such as on-line food stores and restaurants can be alternative sources of foods sold. Considering the growing importance of these food sources, future reviews should take them into account. In addition, this review deliberately focused on interventions promoting the consumption of healthy food (or discouraging unhealthy food) in store settings, whereas effectiveness of interventions reported by marketing (mostly non-food) research was not evaluated.

Studies often vary with regard to their design, methodological quality, settings, population studied, and the intervention, test, or condition considered [94]. Even a study rated best currently may be challenged over time [95]. Besides, most studies used a single intervention store. To increase external validity, and hence methodological quality of the future studies, multiple intervention stores as well as control stores are needed.

Although we were careful in selecting key-words and databases for literature research, it is possible that not all relevant studies are detected. Furthermore, some studies that scored low in the methodological quality may have other strengths not accounted for by our scoring system. Despite these limitations our study was rigorous and systematic.

There are also methodological challenges that are not unique to food environment research. On the one hand, reliability of food frequency questionnaires [53, 63, 75, 78] to measure consumption of healthy foods can be questioned due to over- or underreporting. On the other hand, using sales data to judge effectiveness of interventions, assumes that quantity purchased is equal to quantity consumed. Although objective sales data may provide a fairly accurate approximation to consumption, their validity could perhaps be enhanced by supplementing them with food frequency questionnaires, and comparing the two.

With regard to future research considerations, more studies with randomized controlled trials design with sufficient sample size (both in terms of targeted stores and individual customers) are required to ensure high quality of studies. Most of the reviewed studies have relatively small sample size for their analysis. Future studies should try to fill this gap by using larger sample sizes to ensure their external validity.

Despite the increasing popularity of nudging, there are currently not many food store intervention studies that test the effect of choice architecture on the sales performance of healthy foods. For example, few studies demonstrated effect of using shelf space management to promote healthy foods in prime in-store locations [58, 73, 80]. It is particularly interesting as some prime locations like the checkout area are currently used for promoting high calorie foods. As shown by Sigurdsson et al. [58, 96], these can be replaced with healthful foods. Therefore, more experiments with nudging and other innovative intervention methods in grocery settings are needed. Besides, more focus should be given to both healthy and unhealthy foods and substitution behavior. The majority of current interventions focus on F&V as the promoted healthy food. While these interventions are rightly justified as most people in many countries do not meet F&V dietary guidelines, there is also a need to consider interventions to limit the consumption of less healthy foods, e.g. high energy items such as sugar sweetened beverages (SSB) and salty snacks [92]. If possible, total food store sales should be used to judge the overall effect of the intervention (including substitution effects). Although differences in studies are unavoidable and understandable, adopting some common outcome measures would be useful to enhance comparability of studies. Moreover, food frequency questionnaires used in some studies, if possible, should be supplemented with objective sales data.

Policy decisions are based on the cost-effectiveness of projects, but the literature lags behind when it comes to cost-effectiveness analysis of food store interventions. Sacks et al. [38] is the only study (not included in the review as it did not meet the inclusion criteria) we found that looked at the cost-effectiveness of one of the intervention strategies considered in this review, and they concluded that ‘traffic-light’ nutrition labeling is a cost-effective strategy from the perspective of society. Further studies on the cost-effectiveness of alternative store-setting strategies are definitely needed to help policy makers’ decisions.

## Conclusion

In this systematic review, we assessed the effectiveness and methodological quality of various interventions in food store settings. Given the diverse study settings and despite the challenges of low methodological quality in some studies, we find efficacy of in-store/point-of-purchase healthy food interventions. Increase in purchase and consumption of healthy foods reported by the majority of the reviewed studies, including some with high methodological quality, indicates that in-store intervention strategies may hold a promise in the fight against obesity. Nevertheless, there is need for more high quality studies in food store settings. Our findings also highlight the challenges involved in in-store healthy food interventions. We cannot stress enough the importance of stakeholder management and use of right incentives for these agents, particularly the food stores whose support is critical for any effort in this direction. Most interventions used a combination of information (e.g. awareness raising through food labeling, promotions, campaigns, etc.) and making healthy food available for consumers. Few used price interventions. All in all, interventions which combine price, information and easy access to and availability of healthy foods with interactive and engaging nutrition information, if carefully designed can help customers of food stores to buy and consume more healthy foods. Policy makers should pay special attention to the effect of price incentives on consumer behavior. As has been shown by several randomized controlled trials, price incentives contribute significantly to the effectiveness of intervention strategies, especially when combined with other components such as nutrition knowledge. Such information is useful for the design of intervention instruments that make eating healthier food options attractive while at the same time making unhealthy food the less attractive choice.

## Additional files

Additional file 1: Appendix 1. Search terms used. Contains search terms used for database literature search. (DOCX 15 kb)
Additional file 2: Appendix 2. Summary of methodological quality scores. Contains a table showing the methodological assessment scores for the reviewed studies. (DOCX 93 kb)

## Abbreviations

BHS: Baltimore healthy stores
BMI: Body mass index
F&V: Fruit and vegetables
POP: Point-of-purchase
PRISMA: Preferred reporting items for systematic reviews and meta-analyses
QATSDD: A 16-item quality qssessment tool
RCT: Randomized controlled trial
SD: Standard deviation
SSB: Sugar sweetened beverages
WIC: Women, infants and children
