# Supplementary material for: What is the effectiveness of obesity related interventions at retail grocery stores and supermarkets? —a systematic review
Source: BMC Public Health. 2016 Dec 28;16:1247. doi: 10.1186/s12889-016-3985-x (PMC5192566; doi:10.1186/s12889-016-3985-x)
Supplement: Additional file 1: Appendix 1. — Search terms used. Contains search terms used for database literature search. (DOCX 15 kb) [file 12889_2016_3985_MOESM1_ESM.docx]

# Appendix 1

| Box 1. ***Search terms used*** |
| --- |
| - “Point of purchase” AND “intervention” AND “obesity” - “Obesity” AND “supermarket intervention” - “Obesity” AND “store intervention” - “Nutrition” AND “supermarket intervention” - “Nutrition” AND “store intervention” - “Obesity” AND “environmental intervention” AND “food store” - “BMI” AND “food store intervention” - “BMI” AND “food store” AND “health intervention” - “Obesity intervention” AND “grocery store” - “Obesity intervention” AND “food store” - “Obesity intervention” AND “supermarket” - “Obesity” AND “price/discount” AND “grocery store” - “Obesity” AND “price/discount” AND “supermarket” - “Obesity” AND “promotion” AND “food store” - “Obesity” AND “promotion” AND “supermarket” |
