# Supplementary material for: What is the effectiveness of obesity related interventions at retail grocery stores and supermarkets? —a systematic review
Source: BMC Public Health. 2016 Dec 28;16:1247. doi: 10.1186/s12889-016-3985-x (PMC5192566; doi:10.1186/s12889-016-3985-x)
Supplement: Additional file 2: Appendix 2. — Summary of methodological quality scores. Contains a table showing the methodological assessment scores for the reviewed studies. (DOCX 93 kb) [file 12889_2016_3985_MOESM2_ESM.docx]

Appendix 2: Summary of methodological quality scores

| Score (0-3) | | | | | | | | | | | | |  |
| --- | --- | --- | --- | --- | --- | --- | --- | --- | --- | --- | --- | --- | --- |
| Criteria | Waterlander et al.^(^[^26^](#_ENREF_26)^)^ | Bihan et al.^(^[^57^](#_ENREF_57)^)^ | Mhurchu et al.^(^[^60^](#_ENREF_60)^)^ | Mhurchu et al.^(^[^61^](#_ENREF_61)^)^ | Blakely et al.^(^[^59^](#_ENREF_59)^)^ | Kennedy et al.^(^[^50^](#_ENREF_50)^)^ | Ayala et al.^(^[^66^](#_ENREF_66)^)^ | Dannefer et al.^(^[^72^](#_ENREF_72)^)^ | Steenhuis et al.^(^[^63^](#_ENREF_63)^)^ | Steenhuis et al.^(^[^62^](#_ENREF_62)^)^ | An et al.^(^[^23^](#_ENREF_23)^)^ | Song et al.^(^[^83^](#_ENREF_83)^)^ | Sigurdsson et al.^(^[^58^](#_ENREF_58)^)^ |
| Explicit theoretical framework | 3 | 2 | 2 | 1 | 2 | 1 | 1 | 2 | 3 | 2 | 1 | 3 | 1 |
| Statement of aims/objectives in main body of report | 3 | 3 | 3 | 3 | 3 | 3 | 3 | 3 | 3 | 3 | 3 | 3 | 3 |
| Clear description of research setting | 3 | 3 | 3 | 3 | 3 | 3 | 3 | 3 | 3 | 3 | 3 | 3 | 3 |
| Evidence of sample size considered in terms of analysis | 3 | 3 | 3 | 1 | 1 | 0 | 3 | 0 | 0 | 0 | 0 | 0 | 0 |
| Representative sample of target group of a reasonable size | 1 | 2 | 3 | 1 | 3 | 1 | 2 | 2 | 3 | 2 | 3 | 1 | 1 |
| Description of procedure for data collection | 3 | 3 | 3 | 3 | 3 | 3 | 3 | 3 | 3 | 3 | 3 | 2 | 3 |
| Rationale for choice of data collection tool(s) | 3 | 3 | 3 | 3 | 3 | 2 | 2 | 1 | 3 | 2 | 3 | 2 | 1 |
| Detailed recruitment data | 2 | 2 | 3 | 3 | 3 | 2 | 3 | 1 | 3 | 3 | 3 | 2 | 1 |
| Statistical assessment of reliability and validity of measurement tool(s)  (Quantitative only) | 2 | 1 | 2 | 2 | 0 | 2 | 0 | 0 | 2 | - | 3 | - | 0 |
| Fit between stated research question and method of data collection  (Quantitative only) | 3 | 3 | 3 | 3 | 3 | 3 | 3 | 2 | 3 | - | 2 | - | 2 |
| Fit between stated research question and format and content of data collection tool e.g. interview schedule  (Qualitative only) | - | - | - | - | - | 3 | 3 | - | 3 | 3 | - | 2 | - |
| Fit between research question and method of analysis  (Quantitative only) | 3 | 3 | 3 | 1 | 3 | 2 | 2 | 2 | 2 | - | 3 | - | 2 |
| Good justification for analytic method selected | 3 | 2 | 2 | 1 | 1 | 0 | 2 | 1 | 3 | 0 | 3 | 1 | 0 |
| Assessment of reliability of analytic process  (Qualitative only) | - | - | - | - | - | 2 | 1 | - | 3 | 0 | - | 0 | - |
| Evidence of user involvement in design | 1 | 1 | 3 | 0 | 3 | 0 | 0 | 0 | 3 | 3 | 0 | 1 | 3 |
| Strengths and limitations critically discussed | 2 | 3 | 3 | 3 | 3 | 0 | 3 | 2 | 3 | 0 | 3 | 3 | 0 |
| Score total/maximum score possible | 35/42 | 34/42 | 39/42 | 28/42 | 34/42 | 27/48 | 34/48 | 22/42 | 43/48 | 24/42 | 33/42 | 23/42 | 20/42 |

| Score (0-3) | | | | | | | | | | | | |  |
| --- | --- | --- | --- | --- | --- | --- | --- | --- | --- | --- | --- | --- | --- |
| Criteria | Sutherland et al.^(^[^77^](#_ENREF_77)^)^ | Herman et al.^(^[^29^](#_ENREF_29)^)^ | Herman et al.^(^[^28^](#_ENREF_28)^)^ | Geliebter et al.^(^[^24^](#_ENREF_24)^)^ | Cummins et al.^(^[^55^](#_ENREF_55)^)^ | Colapinto and Malaviarachchi^(^[^52^](#_ENREF_52)^)^ | Milliron et al.^(^[^81^](#_ENREF_81)^)^ | Rosecrans et al.^(^[^54^](#_ENREF_54)^)^ | Novotny et al.^(^[^82^](#_ENREF_82)^)^ | Gittelsohn et al.^(^[^65^](#_ENREF_65)^)^ | Gittelsohn et al.^(^[^97^](#_ENREF_97)^)^ | Gittelsohn et al.^(^[^74^](#_ENREF_74)^)^ | Foster et al.^(^[^73^](#_ENREF_73)^)^ |
| Explicit theoretical framework | 2 | 2 | 2 | 3 | 1 | 1 | 1 | 2 | 3 | 3 | 3 | 3 | 2 |
| Statement of aims/objectives in main body of report | 3 | 3 | 3 | 3 | 3 | 3 | 3 | 3 | 3 | 2 | 3 | 3 | 3 |
| Clear description of research setting | 3 | 3 | 3 | 2 | 3 | 3 | 3 | 3 | 3 | 3 | 3 | 3 | 3 |
| Evidence of sample size considered in terms of analysis | 0 | 0 | 1 | 3 | 1 | 3 | 3 | 0 | 0 | 0 | 0 | 0 | 0 |
| Representative sample of target group of a reasonable size | 3 | 2 | 2 | 2 | 2 | 2 | 1 | 1 | 2 | 2 | 1 | 1 | 1 |
| Description of procedure for data collection | 3 | 3 | 3 | 3 | 3 | 3 | 3 | 3 | 3 | 3 | 3 | 3 | 3 |
| Rationale for choice of data collection tool(s) | 3 | 3 | 3 | 3 | 2 | 1 | 3 | 2 | 2 | 2 | 2 | 2 | 3 |
| Detailed recruitment data | 0 | 2 | 3 | 3 | 3 | 2 | 3 | 1 | 2 | 1 | 1 | 2 | 2 |
| Statistical assessment of reliability and validity of measurement tool(s)  (Quantitative only) | 1 | 0 | 3 | 3 | 2 | 0 | 3 | 0 | 0 | 0 | 0 | 0 | 1 |
| Fit between stated research question and method of data collection  (Quantitative only) | 1 | 2 | 2 | 3 | 2 | 1 | 2 | 2 | 2 | 1 | 2 | 2 | 3 |
| Fit between stated research question and format and content of data collection tool e.g. interview schedule  (Qualitative only) | - | 1 | 1 | - | - | - | - | 3 | 2 | 2 | - | 2 | - |
| Fit between research question and method of analysis  (Quantitative only) | 1 | 1 | 2 | 3 | 2 | 1 | 3 | 2 | 2 | 2 | 2 | 3 | 3 |
| Good justification for analytic method selected | 2 | 0 | 2 | 1 | 3 | 0 | 3 | 2 | 0 | 0 | 2 | 2 | 2 |
| Assessment of reliability of analytic process  (Qualitative only) | - | 1 | 1 | - | - | - | - | 1 | 1 | 1 | - | 1 | - |
| Evidence of user involvement in design |  | 0 | 0 | 0 | 1 | 1 | 1 | 2 | 2 | 2 | 1 | 2 | 3 |
| Strengths and limitations critically discussed | 2 | 1 | 3 | 3 | 3 | 2 | 3 | 2 | 1 | 3 | 3 | 3 | 3 |
| Score total/maximum score possible | 24/42 | 24/48 | 34/48 | 35/42 | 31/42 | 23/42 | 35/42 | 29/48 | 28/48 | 27/48 | 26/42 | 32/48 | 32/42 |

| Score (0-3) | | | | | | | | | | | | |  |
| --- | --- | --- | --- | --- | --- | --- | --- | --- | --- | --- | --- | --- | --- |
| Criteria | Freedman and Connors^(^[^69^](#_ENREF_69)^)^ | Ogawa et al.^(^[^56^](#_ENREF_56)^)^ | Holmes et al.^(^[^80^](#_ENREF_80)^)^ | Freedman et al.^(^[^68^](#_ENREF_68)^)^ | Andrejeva et al.^(^[^70^](#_ENREF_70)^)^ | Caldwell et al.^(^[^67^](#_ENREF_67)^)^ | Song et al.^(^[^76^](#_ENREF_76)^)^ | Ho et al.^(^[^53^](#_ENREF_53)^)^ | Bains et al.^(^[^51^](#_ENREF_51)^)^ | Gittelsohn et al.^(^[^75^](#_ENREF_75)^)^ | Gittelsohn et al.^(^[^78^](#_ENREF_78)^)^ | Curran et al.^(^[^71^](#_ENREF_71)^)^ | Phipps et al.^(^[^25^](#_ENREF_25)^)^ |
| Explicit theoretical framework | 1 | 1 | 2 | 1 | 1 | 2 | 2 | 2 | 2 | 1 | 2 | 3 | 1 |
| Statement of aims/objectives in main body of report | 3 | 2 | 3 | 3 | 3 | 3 | 3 | 3 | 3 | 3 | 2 | 3 | 3 |
| Clear description of research setting | 3 | 3 | 3 | 3 | 3 | 3 | 3 | 3 | 3 | 3 | 3 | 3 | 3 |
| Evidence of sample size considered in terms of analysis | 0 | 0 | 0 | 0 | 2 | 1 | 0 | 0 | 0 | 3 | 0 | 0 | 0 |
| Representative sample of target group of a reasonable size | 2 | 1 | 1 | 2 | 2 | 2 | 2 | 1 | 1 | 1 | 1 | 1 | 1 |
| Description of procedure for data collection | 2 | 3 | 3 | 3 | 3 | 3 | 3 | 3 | 3 | 3 | 3 | 2 | 3 |
| Rationale for choice of data collection tool(s) | 0 | 1 | 2 | 1 | 3 | 2 | 2 | 2 | 2 | 2 | 1 | 2 | 2 |
| Detailed recruitment data | 0 | 0 | 0 | 2 | 0 | 2 | 2 | 0 | 3 | 2 | 2 | 1 | 3 |
| Statistical assessment of reliability and validity of measurement tool(s)  (Quantitative only) | 1 | 1 | 2 | 1 | 3 | 1 | 0 | 0 | 1 | 0 | 0 | 0 | 0 |
| Fit between stated research question and method of data collection  (Quantitative only) | 1 | 1 | 1 | 2 | 2 | 2 | 1 | 2 | 1 | 2 | 2 | 1 | 1 |
| Fit between stated research question and format and content of data collection tool e.g. interview schedule  (Qualitative only) | - | - | 2 | 3 | - | - | 3 | 2 | - | 2 | 2 | - | 2 |
| Fit between research question and method of analysis  (Quantitative only) | 2 | 2 | 2 | 3 | 3 | 2 | 2 | 2 | 2 | 3 | 3 | 2 | 2 |
| Good justification for analytic method selected | 0 | 0 | 0 | 2 | 2 | 1 | 0 | 1 | 1 | 0 | 1 | 1 | 2 |
| Assessment of reliability of analytic process  (Qualitative only) | - | - | 2 | 0 | - | - | 2 | 1 | - | 2 | 0 | - | 0 |
| Evidence of user involvement in design | 0 | 0 | 2 | 2 | 0 | 0 | 1 | 0 | 3 | 3 | 3 | 1 | 2 |
| Strengths and limitations critically discussed | 3 | 3 | 0 | 3 | 3 | 2 | 3 | 2 | 2 | 2 | 3 | 2 | 2 |
| Score total/maximum score possible | 18/42 | 18/42 | 25/48 | 31/48 | 30/42 | 26/42 | 29/48 | 24/48 | 27/42 | 32/48 | 28/48 | 22/42 | 27/48 |

|  | Score (0-3) | | | | | | | | | | | | |
| --- | --- | --- | --- | --- | --- | --- | --- | --- | --- | --- | --- | --- | --- |
| Criteria | Salmon et al., 2015^(^[^64^](#_ENREF_64)^)^ | Ball et al., 2015^(^[^27^](#_ENREF_27)^)^ | Martínez-Donate et al., 2015^(^[^79^](#_ENREF_79)^)^ |  |  |  |  |  |  |  |  |  |  |
| Explicit theoretical framework | 2 | 1 | 3 |  |  |  |  |  |  |  |  |  |  |
| Statement of aims/objectives in main body of report | 3 | 3 | 3 |  |  |  |  |  |  |  |  |  |  |
| Clear description of research setting | 3 | 3 | 3 |  |  |  |  |  |  |  |  |  |  |
| Evidence of sample size considered in terms of analysis | 2 | 3 | 1 |  |  |  |  |  |  |  |  |  |  |
| Representative sample of target group of a reasonable size | 2 | 3 | 1 |  |  |  |  |  |  |  |  |  |  |
| Description of procedure for data collection | 3 | 3 | 3 |  |  |  |  |  |  |  |  |  |  |
| Rationale for choice of data collection tool(s) | 3 | 3 | 1 |  |  |  |  |  |  |  |  |  |  |
| Detailed recruitment data | 3 | 3 | 3 |  |  |  |  |  |  |  |  |  |  |
| Statistical assessment of reliability and validity of measurement tool(s)  (Quantitative only) | 0 | 3 | 1 |  |  |  |  |  |  |  |  |  |  |
| Fit between stated research question and method of data collection  (Quantitative only) | 3 | 3 | 2 |  |  |  |  |  |  |  |  |  |  |
| Fit between stated research question and format and content of data collection tool e.g. interview schedule  (Qualitative only) | - | - | 2 |  |  |  |  |  |  |  |  |  |  |
| Fit between research question and method of analysis  (Quantitative only) | 3 | 3 | 2 |  |  |  |  |  |  |  |  |  |  |
| Good justification for analytic method selected | 1 | 2 | 1 |  |  |  |  |  |  |  |  |  |  |
| Assessment of reliability of analytic process  (Qualitative only) | - | - | 0 |  |  |  |  |  |  |  |  |  |  |
| Evidence of user involvement in design | 0 | 2 | 1 |  |  |  |  |  |  |  |  |  |  |
| Strengths and limitations critically discussed | 1 | 3 | 2 |  |  |  |  |  |  |  |  |  |  |
| Score total/maximum score possible | 29/42 | 38/42 | 29/48 |  |  |  |  |  |  |  |  |  |  |
